# Phylogenetics in the Bioinformatics Culture of Understanding

**DOI:** 10.1002/cfg.381

**Published:** 2004-03

**Authors:** Robin G. Allaby, Mathew Woodwark

**Affiliations:** EST Bioinformatics AstraZeneca Alderley Park Macclesfield Cheshire SK10 4TG UK

## Abstract

Bioinformatics, as a relatively young discipline, has grown up in a world of high-throughput large volume data that requires automatic analysis to enable us to stay on top of it all. As a response, the bioinformatics discipline has developed strategies to find patterns in a ‘low signal : noise ratio’ environment. While the need to process large amounts of information and extract hypotheses is both laudable and inescapable, the pressures that such requirements have introduced can lead to short cuts and misapprehensions. This is particularly the case with reference to assumptions about the underlying evolutionary theories that are implicitly invoked by the algorithms utilised in the analysis pipelines. The classic example is the misuse of the term ‘homologous’ to mean ‘similar’ or even ‘functionally similar’, rather than the correct definition of ‘having the same evolutionary origin’, which may or may not imply similarity of function. In this review, we outline some of the common phylogenetic questions from a bioinformatics perspective that can be better addressed with a deeper understanding of evolutionary principles and show, with examples from the amidohydrolase and Toll families, that quite different conclusions can be drawn if such approaches are taken. This review focuses on the importance of the underlying evolutionary biology, rather than assessing the merits of different phylogenetic techniques. The relative merits of *a priori* and *a posteriori* inclusion of biological information are discussed.


**Comparative and Functional Genomics**
*Comp Funct Genom* 2004; **5**: 128–146.
Published online in Wiley InterScience (www.interscience.wiley.com). **DOI:** 10.1002/cfg.381

**Review**

### Phylogenetics in the bioinformatics culture of understanding

Robin G. Allaby and Mathew Woodwark*
*EST Bioinformatics, AstraZeneca, Alderley Park, Macclesfield, Cheshire SK10 4TG, UK*

**Correspondence to: Mathew Woodwark, EST Bioinformatics, AstraZeneca, Alderley Park, Macclesfield, Cheshire SK10 4TG, UK. E-mail: Mathew.Woodwark@astrazeneca.com*

Received: 18 July 2003
Revised: 10 December 2003
Accepted: 22 December 2003

### Abstract

**Bioinformatics, as a relatively young discipline, has grown up in a world of high-throughput large volume data that requires automatic analysis to enable us to stay on top of it all. As a response, the bioinformatics discipline has developed strategies to find patterns in a 'low signal : noise ratio' environment. While the need to process large amounts of information and extract hypotheses is both laudable and inescapable, the pressures that such requirements have introduced can lead to short cuts and misapprehensions. This is particularly the case with reference to assumptions about the underlying evolutionary theories that are implicitly invoked by the algorithms utilised in the analysis pipelines. The classic example is the misuse of the term 'homologous' to mean 'similar' or even 'functionally similar', rather than the correct definition of 'having the same evolutionary origin', which may or may not imply similarity of function. In this review, we outline some of the common phylogenetic questions from a bioinformatics perspective that can be better addressed with a deeper understanding of evolutionary principles and show, with examples from the amidohydrolase and Toll families, that quite different conclusions can be drawn if such approaches are taken. This review focuses on the importance of the underlying evolutionary biology, rather than assessing the merits of different phylogenetic techniques. The relative merits of *a priori* and *a posteriori* inclusion of biological information are discussed. Copyright © 2004 John Wiley & Sons, Ltd.**

**Keywords:** phylogenetics; evolutionary tinkering; amidohydrolase; Toll; atrazine

### Introduction

In recent years, the development of bioinformatics has been influenced heavily by the explosion of biological data in the public domain. There are about 19 complete eukaryote genomes currently available from principal public domains, as well as 89 bacterial, 15 archaeal and 1001 viral genomes (**http://www.ncbi.nlm.nih.gov/entrez/query.fcgi?db=Genome**). The list of current genome sequencing projects suggests that the eukaryote genome archive will soon double in size and, in particular, the vertebrate section of that archive is set to become over three times its current size.

In order to extract meaningful information from such vast repositories of sequence data the bioinformatics discipline has developed strategies to find patterns in a 'low signal : noise ratio' environment. The efforts to produce successful analyses through the development and refinement of BLAST and profile searching approaches has resulted in an established bioinformatics culture of understanding, to which we will refer in this article as a '*sensu bifo*'. The *sensu bifo* now has a principal role in shaping how analyses are approached.

In the post-genomic era we are moving to a deeper *in silico* understanding of biology with bioinformatics goals including the assignment of function to the whole of the genomic blueprint. The extent of data coverage has now reached the stage at which one can judge the apparent omission of one gene in one genome to be the result of a real biological process rather than simply a case of missing data. Consequently, there has been an

Copyright © 2004 John Wiley & Sons, Ltd.

increasing tendency to ask questions of a phylogenetic nature. The most frequent enquiries appear to concern orthologous relationships between genes. Often workers wish to know which gene of a genome is the orthologue of a well-characterized gene in another genome, in order to be able to infer something about the function of the new gene. In the drug discovery process, this stage may help to predict which parts of the uncharacterized genome are likely to be useful targets for drug development. However, it is also frequently the case that bioinformaticians find difficulties with phylogenetic analysis, reaching an apparent impasse at an early stage. Often only very poor alignments are generated, which give rise to inconclusive trees, or there is an apparent total absence of gene sequences that could potentially be orthologous. In such situations there appears to be no obvious answer.

In this article we address some of the issues of how a phylogenetic analysis should be approached and interpreted by bioinformaticians who have little previous experience in the subject area. It will become apparent that the correct application of such approaches can lead to quite different conclusions from an initial stereotypical *sensu bifo*-based approach. It will be shown in this paper that an understanding of the evolutionary process is necessary in order to answer phylogenetic questions, and should be a principal force in shaping the approach to analysis. In many cases, adoption of a practice more firmly based in evolutionary biology will result in the negation of apparent analytical impasses. It is the intention of this paper to focus on the importance of the evolutionary biology concerned rather than the specific type of phylogenetic algorithm applied to an analysis.

### Evolutionary processes that complicate phylogenetics

Obviously, there are many evolutionary processes in nature and it is not the remit of this article to attempt to review them all. The discussion will be restricted to some points particularly pertinent to the drug discovery process. Specifically, this article will deal with the subject area of mobile domains and how the various aspects of this evolutionary phenomenon occur and can be incorporated into phylogenetic analyses.

Many of the genes that are of interest to the drug discovery process code for proteins that are either transmembrane or completely extracellular. Pharmacologically attractive gene families which fit these categories include the G protein-coupled receptors (GPCRs) and their ligands, on which half of all modern drugs act (Howard *et al.*, 2001). There is also interest in other transmembrane receptor groups, such as the *Toll*-like receptors, which have been implicated in the innate immune response (reviewed in Hoffman *et al.*, 1999; Zuany-Amorim *et al.*, 2002). An understanding of the evolutionary history of these genes will allow us to facilitate the process of function assignment to previously uncharacterized family members.

### Modular genes

The majority of extracellular and transmembrane proteins are encoded by modular genes (Patthy, 1999). Moreover, the human genome as a whole contains an alarming number of genes that share domains with other genes, also implying the presence of modular genes (Li *et al.*, 2001). Modular genes are ones that are comprised of a combination of modular domains obtained from different sources. The types of module domain range from the very generic and widespread, e.g. the serine protease domain, to the more specific domains, such as the Toll and interleukin (IL)–1 receptor (TIR) domains exemplified in the *Toll*-like receptors outlined in the second of our case histories below. It appears likely that the formation of modular genes may also be important in the evolution of pathways of protein–protein interactions, as outlined by the Rosetta Stone model (Marcotte *et al.*, 1999). Consequently, a modular gene can represent a mosaic of evolutionary history, each module of which could be better understood by drawing separate phylogenetic trees.

### Evolutionary 'tinkering'

The occurrence of modular genes in nature gives the potential for a vast array of new genes because modules can be used and reused, just as a computer programmer can use and reuse objects in different contexts. The concept of modular genes leads naturally to the concept of 'evolutionary tinkering', described by Jacob (1977). The concept of tinkering emphasizes that evolution progresses through

Copyright © 2004 John Wiley & Sons, Ltd. 

the use and reuse of genetic 'machinery' that is already in existence, even though that machinery may not be optimal for the task. It is an important aspect of the tinkering concept that the resulting genetic machinery does not necessarily reflect the optimal solution. Nature is littered with examples of suboptimal evolutionary solutions. Classically, at the whole organism level, there is the example of the bee sting that, suboptimally, kills the bee. At the molecular level there is example of the rubisco protein that has a suboptimal affinity for $CO_2$, but also, more disastrously for the plant, a significant affinity for $O_2$, leading to the energetically wasteful process of photorespiration. A strikingly successful result of tinkering in terms of diversity appears to be the evolution of the GPCR family (Bockaert and Pin, 1999). The GPCR family represents an extremely diverse group of proteins capable of signal transduction across a cell membrane using cues ranging from light through to $Ca^{2+}$ to odorants. The GPCR family also illustrates the fact that recombination plays an important role in the tinkering process (Shields, 2000). Tinkering can lead to quite unexpected domains occurring together, as in the case of the *Sdic* gene in *Drosophila melanogaster*, which is the result of the fusion of part of the *AnnX* gene with part of the *Cdic* gene (Nurminsky *et al*., 1998), where the parent genes are quite unlike the hybrid progeny gene. In this particular case the sequence that was an intron in the *Cdic* gene became an exon in the *Sdic* gene. A natural consequence of the tinkering process is that prediction of the function of a protein based on the function of an apparently evolutionarily close neighbour is not necessarily secure. This aspect is exemplified in the case history of amidohydrolase proteins, outlined below.

### Evolutionary convergence

The use and reuse of old domains in new contexts in the tinkering process leads one to suppose that some domains will be better suited for reapplication in certain contexts than others. In turn, this leads to another important concept, that of evolutionary convergence and parallelisms, and the stepwise adaptive landscape. The stepwise adaptive landscape can best be imagined as a normal landscape, with normal topography, such as hills and valleys. The hills represent adaptive fitness of the organism as a whole, and any point on the landscape represents a single phenotype, resulting from a specific genotype. The landscape has footpaths on it, representing legal routes through it. In evolutionary terms, the paths represent possible routes of change through the landscape. The evolution of a combination of domains giving rise to a certain gene type can be thought of as a point half-way along one of the paths. The existence of this gene may provide new opportunities for potential genes, which may subsequently evolve. It may also be the case that the footpath is 'trodden along' more than once. A nice example of such an adaptive walk is observed in the GPCR family within the chemokine receptor family (Hughes and Yeager, 1999). In this case macrophage inflammatory proteins (MIP) receptors have evolved first, and then a loss of features in the third and fourth domains have given rise to monocyte chemoattractant protein (MCP) receptors. It would appear that the intermediary step of the MIP receptors is required for the evolution of the MCP receptor. However, there are two MIP receptors (CCR1 and CCR5) and two MCP receptors (CCR2 and CCR3), which do not form clades equating to function in a phylogenetic analysis. Indeed, CCR2 forms a clade with CCR5 and CCR3 joins CCR1. Consequently, it appears to be the case that this is an adaptive walk that has occurred on two occasions. One could not infer functionality in this case simply from the position of taxa in a phylogenetic tree.

### Domain 'theft'

The final aspect of domain movement in an evolutionary tinkering context, relates to a more aggressive tinker. It may be the case that the fitness of a genome may be improved by obtaining molecular 'machinery' already evolved in other genomes. The movement of sequence horizontally between genomes is a well-documented and frequently occurring phenomenon. Perhaps one of the more pertinent aspects with regard to the drug discovery process is the apparent ability of pathogens to obtain segments of host genome that appear to play a role in subsequent host invasion by the pathogen. Examples of this include the apparent pilfering of parts of the CCR5 and CXCR4 chemokine receptors by immunodeficiency viruses to aid in the cell invasion process (Shimizu and Gojobori, 2000). In addition to stealing domains from the

Copyright © 2004 John Wiley & Sons, Ltd. 

host genome, pathogens such as viruses can subsequently swap domains between their genomes, resulting in elusion of recognition by the host immune system (Robertson *et al.*, 1995). The fluidity of genes between genomes is also manifested in more benign processes, e.g. the human genome is in receipt of genes and pseudogenes of prokaryotic origin resulting from the degeneration of the mitochondrial genome that can result in the presence of prokaryotic 'molecular fossils' within the nuclear genome (Zischler *et al.*, 1995; Zullo *et al.*, 1991).

### Evolution and phylogenetics

A running theme in the examples of evolutionary processes outlined above is the fact that the data resulting from the evolutionary processes is not necessarily inherently tree-like, but can actually include several tree-like histories. Any approach to a phylogenetic analysis needs to be able to reflect these biological possibilities. Naturally, this leads to the question: how can one incorporate these ideas into a phylogenetic analysis?

It is often the case that at the outset of the analysis we have biological information available over and above the 'raw' sequence data that is pertinent to the evolutionary process. In particular, domain information is increasingly available. A difference in approach regarding *a priori* information can occur at this stage between phylogeneticists and bioinformaticians.

### The blind approach

Frequently, bioinformaticians will take the apparently Fisherian stance that in order for an analysis to remain objective as possible, one should not try and influence an analysis 'unfairly' by trying to incorporate biological preconceptions over and above the algorithms employed in the analysis. In other words, it is more 'scientific' to draw all inferences from the similarity between sequences, so that the results are produced in a blindfold manner. It should be noted that this tendency toward a blindfold approach is congruent with a desire to produce automated large throughput systems that has frequently been the charge of bioinformatics. In describing this as an apparently 'Fisherian' stance, it is intended that the idea of a null hypothesis assuming no *a priori* knowledge is implied. This type of stance implicitly assumes that the algorithms used, e.g. those used for drawing trees, will reflect the biology accurately. The resulting interpretation can consequently be primarily biological rather than mathematical because the two are considered synonymous.

The underlying assumption that phylogenetic algorithms will reflect the biology will be flawed if any of the evolutionary processes outlined in the previous section have occurred. In this particular series of cases, a single phylogenetic tree would be a mathematical representation of the net result of complex biological processes, which are essentially untree-like as a whole. A simplistic interpretation of such a tree is likely to be misleading, as will be outlined in the case histories below.

### Incorporation of a *priori* information

Ideally, a phylogeneticist begins an analysis with *a priori* information in the form of establishing homology between alignments. The analysis can only be sensibly carried out using points of homology between sequences. The preconception that an entire gene is homologous to another, based on the evidence of a BLAST-based result, should not be assumed. A BLAST result might indicate a region or domain that is homologous between two genes, and is extremely useful in that respect, but provides no direct evidence for homology for the rest of the gene. There are numerous biological reasons why the rest of the gene may not be homologous to a true positive BLAST result, some of which are outlined in the previous section.

In the first instance, evidence for homology over the whole gene will come from alignments of the whole gene. A measure of the quality of the alignment can be used to judge whether the relationship is identifiably homologous, and consequently whether the phylogenetic algorithms we use can actually sensibly reconstruct the evolutionary distance between taxa. To assess alignment quality, one can scan the values of a distance matrix produced from a programme designed to account for multiple changes at base or amino acid sites, in order to calculate the evolutionary distance, such as DNADIST or PROTDIST from the PHYLIP package (Felsenstein, 1989). For instance, in our own practice of phylogenetics, threshold values are employed to indicate the level of acceptability of

Copyright © 2004 John Wiley & Sons, Ltd. 

alignments. In the case of nucleic acid distances, a distance of over 1 substitution/base site indicates that the sequence distances are beyond reliable reconstruction. Note that this is considerably more stringent than the 25% sequence similarity that represents two random nucleotide sequences aligned together. A value of around 55% sequence similarity represents 1 base substitution/site, using the simplest model of nucleotide change (Jukes and Cantor, 1969). The value of the number of substitutions plotted against percentage similarity follows an asymptote, increasing rapidly in the lower percentages, reaching 6.69 at 25.01% and 10.15 at 25.0001%. In the case of proteins, the assignation of a threshold value is considerably more subjective. Using a point accepted mutation (PAM) approach, distances are expressed between proteins in terms of the product of substitutions expected for an amino acid composition if the two sequences were 99% similar. Consequently, a PAM-based distance of 1 equates to 100 times the number of substitutions expected if the same sequences were only 1% divergent. A larger number of multiple substitutions can be tolerated for amino acids than for nucleic acids. In the case of nucleotides, the rates at which the different types of base substitution occur tend to be similar, whereas in the case of amino acids the range of substitution rates is very wide. Consequently, amino acid alignments may have a large number of substitutions caused by the high substitution rates of certain sites, while the occurrence of conserved amino acids allows the alignment itself to remain discernible. In practice we use a PAM-based distance of 4 as a warning threshold, based on the observations of Duffy and chemokine sequences. These sequence types share only 25% similarity (Murphy *et al.*, 2000) and at best only share small segments that can actually be considered homologous.

It is often the case in bioinformatics, when examining gene families that span large evolutionary distances, that large portions of the alignment are not recognizably related. This may be due to low sequence conservation combined with a large amount of time having passed since the common ancestor of the sequences, or it might be the case that these sequence regions are really not homologous. In either case, the distance reconstructing algorithms will fail regardless of the biological truth. The domain information present within database entries is good source of *a priori* evidence for homology. The alignment of corresponding domains between sequences helps to ensure homologous comparison, and helps one to avoid trying to align unrelated domains. However, if a single domain is being used in the alignment, the analysis becomes one of that domain rather than the gene as a whole. The story of the gene then becomes the sum of the stories of the domains.

Consequently, in contrast to the bioinformatician, it is the stance of the phylogeneticist to include as much *a priori* information in the shaping of the analysis as is humanly possible. In addition to the domain annotation that may be available from databases, preliminary analyses can be carried out which may help to indicate the occurrence of evolutionary processes involving horizontal domain movement. In some unfortunate cases this may be the only approach available.

### Software for initial analyses

There have been several programmes developed over recent years that are freely available and can help in these initial stages, by analysing the internal consistency of the phylogenetic signal of an alignment. Again, this is not a review of the currently available software, but an indication of types of approach available. There are several approaches to identifying multiple tree topologies within an alignment, and hence recombination events, each of which vary considerably in efficacy and computational expense (Posada and Crandall, 2001). Recombination search methods which employ sliding distance matrices, such as TOPAL (McGuire and Wright 1998), compare favourably and have been shown to be both sensitive and computationally quick. One can view multiple topologies within the data, without assuming a tree-like process, using SplitsTree (Huson, 1998). More recently, efforts have been directed at constructing tree-building packages which allow one to view both the tree and domain structure of the data together, such as in NIFAS (Storm and Sonhammer, 2001). If conflicting tree topologies are found within an alignment, then it may be desirable to qualitatively compare topologies in a tanglegram format, as in TREEMAP (Page, 1995). The tree reconciliation methodologies employed in the latter technology have subsequently and effectively been used to assign orthologous relationships between genes

Copyright © 2004 John Wiley & Sons, Ltd. 

in the context of true species trees (Page, 1998). Finally, one may view the evolutionary history of each base site or amino acid of an alignment in the context of a phylogeny to examine internal congruence, or identify clade-defining sites using character ancestry reconstruction features of the MacClade and Mesquite packages (Maddison and Maddison 2002a, 2002b).

The preliminary stages of analysis using software such as that outlined above will give a good idea of how suitable the data is for phylogenetic analysis, and whether the data should be separated into subalignments, before tree construction is attempted. It may be the case, for instance, that a tree-like process is simply not appropriate to reflect the biology (e.g. Allaby and Brown, 2001).

### Case studies

The following two case studies illustrate how the culture of understanding of bioinformatics leading to the *sensu bifo* can be misleading in a phylogenetic analysis.

### Amidohydrolases

This case history illustrates how the *sensu bifo* can differ from the *sensu stricto* in the definition of evolutionary terms. In this case the difference in definition is with regard to the term 'orthologous'.

Amidohydrolases are a superfamily of enzymes characterized by a common structural architecture, although across the whole family there is low sequence conservation (Holm and Sander, 1997; Copley and Bork, 2000). The structure consists of an ellipsoid $(\beta\alpha)_8$ barrel, with a metal binding site at the C-terminal ends of $\beta_1$, $\beta_5$, $\beta_6$ and $\beta_8$. Typically, amidohydrolases use a metal ion to deprotonate a water molecule that is then used for nucleophilic attack on a substrate. The hydrolytic reactions carried out by amidohydrolases are frequently deamination or dechlorination of small ring molecules, or acting on a small amide ring (Kim and Kim, 1998). It is of little surprise, then, that members of the amidohydrolase superfamily are employed in the purine and pyrimidine catabolism that ultimately results in the production of nitrogenous waste metabolites such as urea and ammonia.

#### Atrazine degradation

Interestingly, the amidohydrolase superfamily includes a group of enzymes that appear to have evolved recently which are involved in the degradation of the herbicide atrazine (Holm and Sander, 1997). Commercially, this is an interesting microbe quality because atrazine can persist in groundwater (Goodrich *et al.*, 1991). Several species of bacteria have been identified that have evolved the ability to utilize atrazine as a nitrogen source, including *Pseudomonas* sp. (strain ADP), *Rhodococcus corallinus* and *Nocardioides* sp. (C190) (Eaton and Karns, 1991; de Souza *et al.*, 1996; Mulbry, 1994; Shao and Behki, 1995; Shao *et al.*, 1995; Topp *et al.*, 2000; Piutti *et al.*, 2003). It would appear that there are two principal metabolic pathways by which the herbicide is degraded, as outlined in Figure 1. The first pathway is carried out by *R. corallinus* and involves the dealkylation of atrazine by cytochrome P450 (Nagy *et al.*, 1995), to give CEAT, followed by dechlorination to produce N-ethylammeline which is deaminated repeatedly to give N-ethylammelide and then cyanuric acid. The alternative pathway, carried out by *Nocardioides* sp. (C190) and *Pseudomonas* sp. (strain ADP), involves the direct dechlorination of the atrazine molecule to produce hydroxyatrazine. The latter is subsequently deaminated to produce N-ethylammelide and then cyanuric acid. Consequently, the two paths converge onto each other in the latter two steps.

#### Orthologue searches

If one submits the *Pseudomonas* sp. (strain ADP) atrazine cholorohydrolase (ATZA) amino acid sequence (Accession No. P72156) as a BLAST search of the public databases, it is quite likely that the functionally corresponding amino acid sequence in *Nocardioides* sp. (C190) (Accession No. Q8VSO1, for the enzyme TRZN) will not be found because it shows only 27% similarity. This leads one to a typical sort of question asked in bioinformatics, which is 'Why can't I find the orthologue of one gene of one species in another species?'.

The query has an inherent simple tree structure associated with it, which is illustrated in Figure 2. It is implicitly assumed that genes of a particular function will be the most closely related. Consequently, species absent from certain clades appear as a glaring and apparently illogical omission.

Copyright © 2004 John Wiley & Sons, Ltd. 

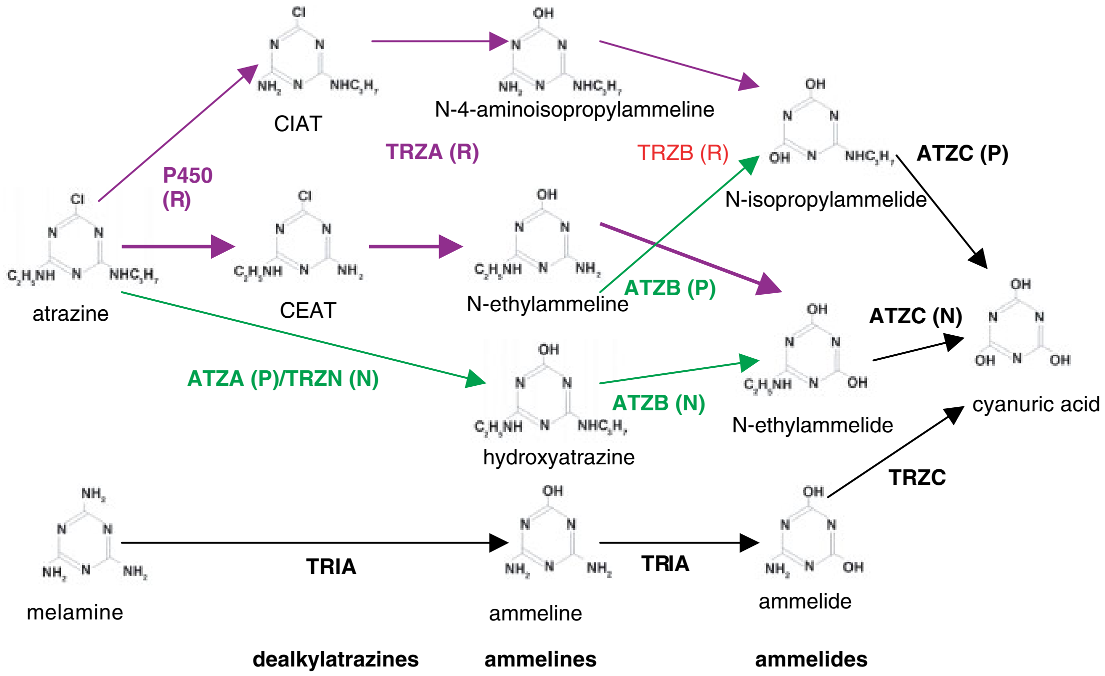

**Figure 1.** Metabolic pathways of atrazine and melamine degradation. Compound names and structures are given. Metabolic pathways are indicated by arrows; proteins responsible for facilitating the reactions are indicated by the arrows; proteins shown in red have not yet had their amino acid sequence determined. The preferred pathways for *Rhodococcus, Pseudomonas* and *Nocardioides* are indicated in brackets using the abbreviations R, P and N respectively. The 'upper' pathway of *Rhodococcus* is described with purple arrows, thicker arrows indicating the principal pathway. The same enzymes carry out the major and minor pathways in *Rhodococcus*. The green arrows describe the 'lower' pathway preferred by *Pseudomonas* and *Nocardioides*. The amino acid sequences of ATZB and ATZC in *Nocardioides* have only recently been partially determined but appear to be 99–100% similar to the corresponding sequences in *Pseudomonas* (Piutti *et al*., 2003). Compiled from Eaton and Karns (1991); de Souza *et al*. (1996); Mulbry (1994); Shao and Behki (1995); Shao *et al*. (1995); Topp *et al*. (2000); Piutti *et al*. (2003); Nagy *et al*. (1995)

An extensive BLAST search quickly leads one to see that the amidohydrolases of the atrazine degradation pathway are most closely related to other amidohydrolases concerned with the hydrolytic reactions involving substrates of guanine, cytosine, adenine, dihydropyrimidine, dihydroorate and imidalazolone-5-propionate.

### Establishing an amidohydrolase alignment

Attempts to include the range of sequences unearthed by the BLAST search in a phylogenetic analysis soon reach an impasse because of the apparent impossibility of getting a good sequence alignment. A distance matrix calculated from the best alignment one can produce from the entire length of

**Figure 2.** An unrooted phenetic tree of amidohydrolases based on an *a priori* assumption of common functionality equating to orthology. This tree was prepared manually to represent the association of function with relatedness. Colour labelling of taxa relates to function: purple, guanine deaminase (GUAD); yellow, imidazolonepropionase (HUTI); turquoise, atrazine chlorohydrolase (ATZA/TRZN); green, N-ethylammeline chlorohydrolase (TRZA); crimson, melamine amidohydrolase (TRIA); grass green, hydroxyatrazine deaminase (ATZB); pink, dihydroorotase (DHOase); red, adenine deaminase (ADND); light blue, dihydropyrmidinase (DHPase); duck egg blue, ammelide amidohydrolase (ATZC/TRZC); faded red, adenosine deaminase (ADSD); lilac, nitrogen fixation protein (NFIX); marine blue, copper binding protein (CUB); sky blue, cytidine deaminase (CTDN); blue, cytosine deaminase (CYTD). Database entries annotated with the function 'chlorohydrolase' without reference to the specific substrate are annotated as CHLY. Branch lengths are not proportional to genetic distance. Species names are given in abbreviated form in all cases except those for which no taxonomic identification below the genus level is given in the database. All trees in this paper were prepared in Mesquite (Maddison and Maddison, 2002b)

Copyright © 2004 John Wiley & Sons, Ltd. 

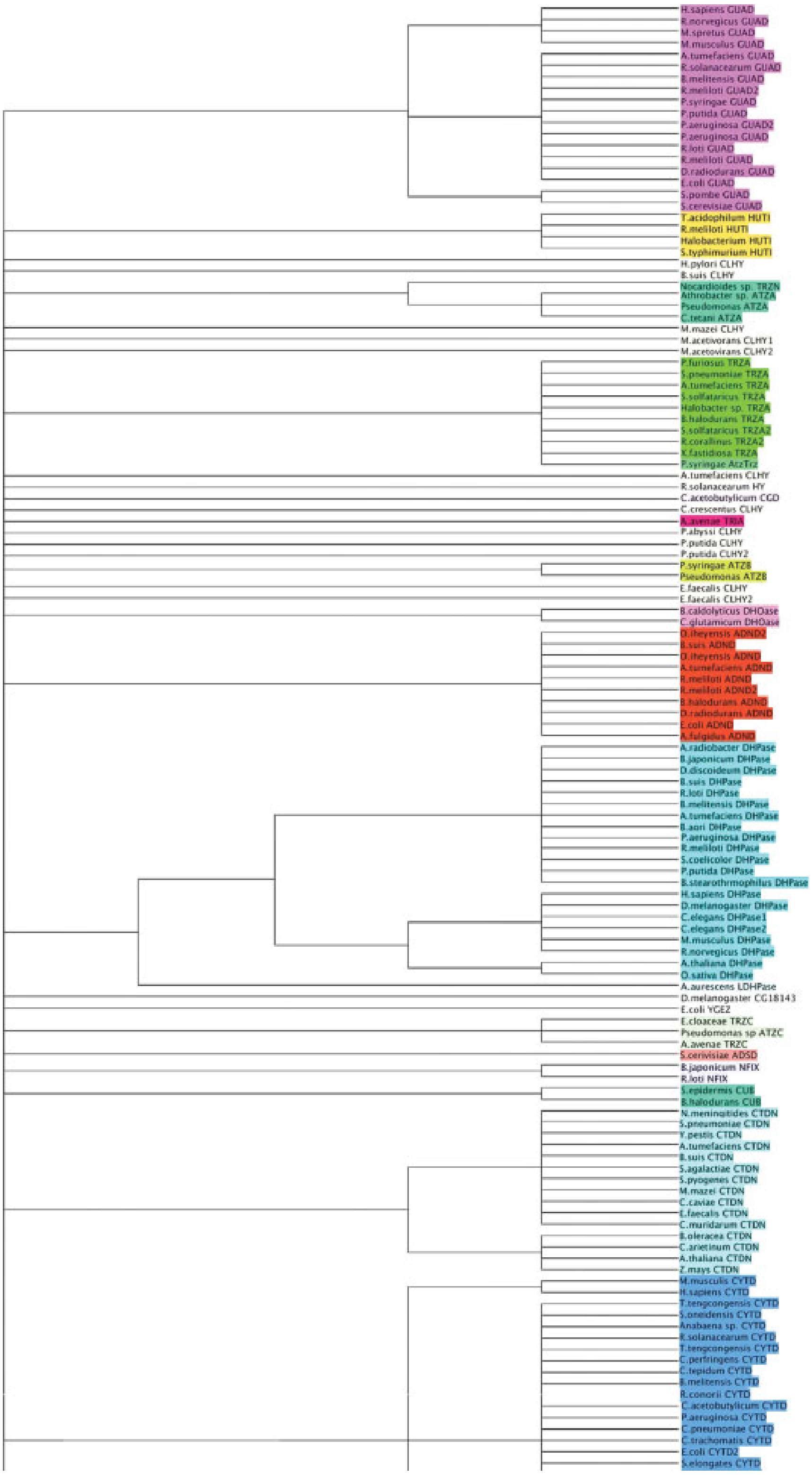

Copyright © 2004 John Wiley & Sons, Ltd.

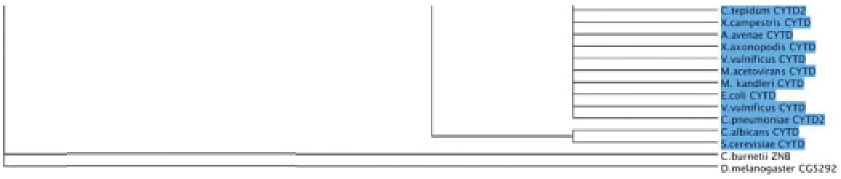

**Figure 2.** Continued amino acids will frequently give distance values (as calculated by PHYLIP's Protdist programme) of over 40. A value of 40 is equivalent to 4000 more amino acid substitutions than one would expect for that amino acid composition if the two taxa under consideration differed at 1% of residues. It is highly doubtful that such a high score could represent an accurate estimate of multiple substitutions between taxa. In addition, as discussed in the previous section, from our *a priori* knowledge of how distant we expect members of a gene family or superfamily to be from each other, again this score seems anomalously (about 10-fold) high. The first objective of the analysis, then, is to establish real points of homology between the taxa.

In this case, sequence conservation has been noted among several groups of amidohydrolases in the N-terminal region (Kim and Kim, 1998; Yuan *et al.*, 1999). Use of this *a priori* information to identify regions that we can be reasonably sure are homologous gives rise to an alignment constructed from the N-terminal region, which has reasonable genetic distances between taxa.

### The phylogenetic trees of amidohydrolases

A tree produced from the acceptable alignment, shown in Figure 3, proves to be enlightening when viewed with an 'evolutionary eye'. The tree as a whole is unrooted; we do not know, *a priori*, which is the oldest part of the tree. Some of the enzyme families form neat monophyletic clades, such as the guanine and adenine deaminases. Notably, however, some enzyme families do not. In particular, the cytosine deaminase family seems particularly diverse. There is a notable absence of animal and plant cytosine deaminases in the tree. In fact, a group of cytosine deaminases that are from a different part of the amidohydrolase superfamily, shown in Figure 4, include sequences from higher eukaryotes as well as prokaryotes. Note that some of the prokaryotes, such as *Vibrio vulnificus*, have sequences of cytosine deaminase in both trees, implying that really quite different proteins are being employed by the organism to carry out similar functions.

The atrazine chlorohydrolase and related atrazine degradation pathway sequences appear to be closely related to cytosine deaminase sequences in Figure 3. Considering the apparent recent evolution of this group of enzymes, it is tempting to speculate that the cytosine deaminase enzymes have been the subject of evolutionary tinkering and applied to the recognition of the triazine ring structures of atrazine, ammeline and ammelide and their derivatives. This possibility is echoed by the fact that the *Pseudomonas* sp. (strain ADP) *S*-triazine catabolic genes were originally suggested to be located on a transposable element (Eaton and Karns, 1991). The relationship between cytosine deaminases and atrazine deaminases is strengthened by the fact that of the substrate preferences of the amidohydrolases present in Figure 3, cytidine is the most similar to the triazines in structure. In fact, it has been observed that the *R. corallinus* enzyme TRZA, so called N-ethylammelide chlorohydrolase, is capable of deaminating the pyrimidines 2,4,6-triaminopyrimidine and 4-chloro-2,6-diaminopyrimidine (Mulbry, 1994).

Enzymes degrading the various atrazine metabolites appear to be paralagous. The gene lineage that gives rise to the ATZA (atrazine hydrochlorinase) genes appears to be paralogous to the ATZB (hydroxyatrazine deaminase) lineage (Boundy-Mills *et al.*, 1997). Similarly, the ATZC (Sadowsky *et al.*, 1998) lineage is paralogous to the ATZA and ATZB lineages. Interestingly, the *Nocardioides* sp. (C190) TRZN (triazine hydrolase) gene responsible for the dechlorination of atrazine appears to be orthologous to the ATZB lineage, not the ATZA lineage. This is not what one might expect if proteins of similar function were to form clades. Consequently, an explanation to the original query, of why a BLAST search with the ATZA sequences did not find the *Nocardioides* sp. (C190) equivalent,

Copyright © 2004 John Wiley & Sons, Ltd. 

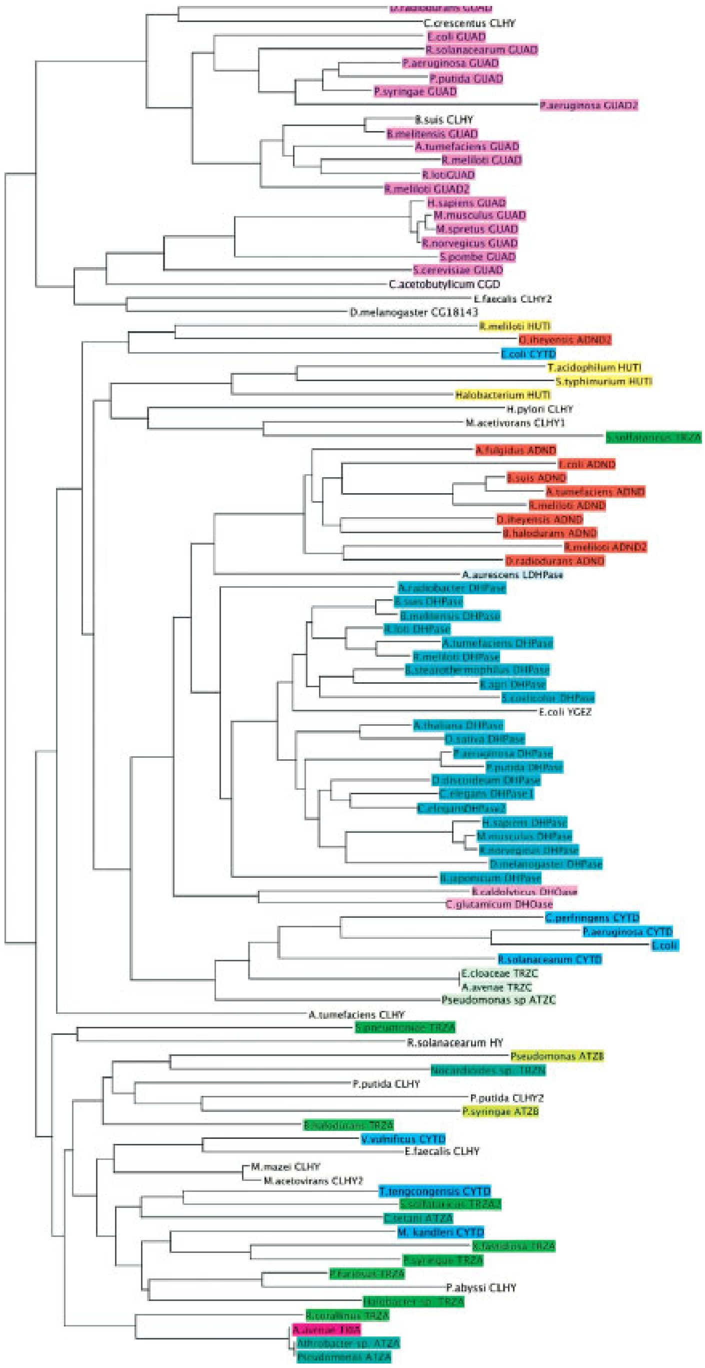

**Figure 3.** An unrooted neighbour-joining tree of conserved region I of amidohydrolases closely related to atrazine degrading amidohydrolases, using the JTT matrix of PROTDIST (Felsenstein, 1989) to calculate distances between taxa. Colour labelling of taxa relates to function; for key, see Figure 1. Branch lengths are proportional to genetic distance. There was an apparent sequence error in the database of the *Rhodococcus corallinus* TRZA gene (Accession No. L16534) of a CA insertion in the nucleotide sequence at positions 91 and 92, giving rise to a frame shift, and a second error between positions 367 and 430, re-establishing the first frame. It is unlikely that insertion is real since the expressed protein has been observed to be functional, whereas the frame shift would cause the conserved region I (Kim and Kim, 1998) to be replaced by a serine/threonine-rich region. The corrected amino acid sequence includes the conserved amino acid residues expected for region I

Copyright © 2004 John Wiley & Sons, Ltd. 

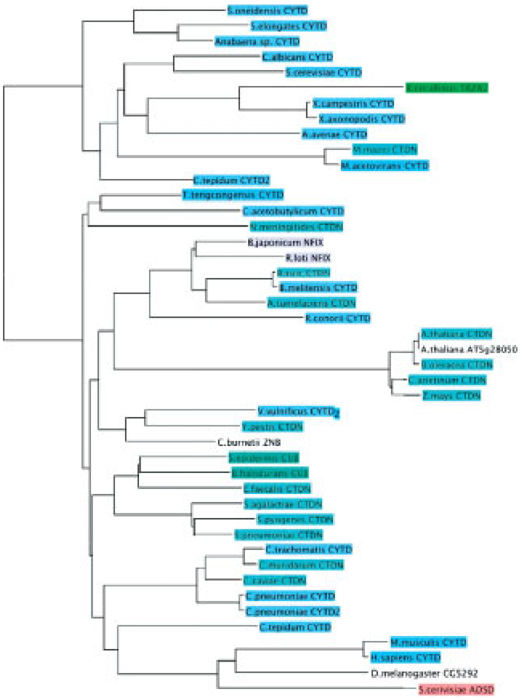

**Figure 4.** An unrooted neighbour-joining tree of amidohydrolases related to cytosine deaminases which are too distant to be aligned accurately to those amidohydrolases of Figure 3. Colour labelling of taxa relates to function; for key, see Figure 1. Branch lengths are proportional to genetic distance appears to lie in the fact that there is a promiscuity of substrate preference in relatively distant lineages. The versatility of substrate preference is also highlighted in this clade by the ability of ATZB enzymes to deaminate or dechlorinate (Seffernick *et al*., 2002).

There are further insights into the evolution of this group of enzymes. The TRZA enzyme of *R. corallinus*, that has been observed to form the second step of the atrazine degradation pathway, appears to be orthologous to ATZA, the enzyme of the first step of the *Pseudomonas* sp. (strain ADP) atrazine degradation pathway. The literature only reports the action of *R. corallinus* TRZA on the dealkylated metabolite of atrazine, 6-chloro-N-ethyl-1,3,5-triazine-2,4-diamine (CEAT). The phylogenetic placement in Figure 3 suggests that this enzyme may have activity on the herbicide directly. The dealkylating ability supplied by cytochrome P450 in *R. corallinus* may serendipitously negate the need for TRZA to act on the alkylated molecule in this organism. This evolutionary tinkering could then have given rise to the second metabolic pathway for atrazine degradation outlines in Figure 1. However, it should be noted that small changes in the sequence have been observed to have quite specific effects on substrate specificity in this group, e.g. melamine deaminase (TriA), which although 98% similar in sequence has no activity on halogenated triazine rings, and ATZA has no activity on melamine (Seffernick *et al*., 2001). Similarly, it is also interesting to note that the isopropylammelide amidohydrolase (ATZC) of *Pseudomonas* sp. (strain ADP) appears to be orthologous to ammelide amidohydrolase (TRZC) of *Acidovorax avenae*, suggesting the possibility of cross-substrate activity.

The final observation relates to the automated annotation of sequences by genome sequencing projects. The only N-ethylammelide hydrochlorinase sequence to be determined empirically in the laboratory is that belonging to *R. corallinus* in Figure 3. A second 'TRZA' *R. corallinus* sequence (labelled *R. corallinus* TRZA2) is present in Figure 4. This sequence is a partial gene sequence also cloned from *R. corallinus*, which has been given the same annotation as the *trzA* gene (Shao *et al*., 1995; Accession No. Q52724). Incredibly, this gene sequence happens to have a high similarity to the group of cytosine deaminases represented in Figure 4, quite different to those of Figure 3. The remaining amino acid sequences that have been quite specifically annotated as N-ethylammelide chlorohydrolase (TRZA) in Figure 3 are the automated result of genome annotation. The slightly deeper understanding of the evolution of this group provided by the tree and the *a priori* information available suggests that is it is highly unlikely that these proteins would be so specific in their substrate preference.

### Orthology is not functional similarity

In summary, the original query assumes a *sensu bifo* definition of the term 'orthologous', that orthologous genes are those that have the same function in different species. Neither the cytosine deaminase genes nor the genes of atrazine degradation are 'orthologous' as groups in the evolutionary definition of the word. In *sensu stricto* an orthologous relationship is one in which two taxa are separated by a speciation event, consequently the phylogenetic tree of orthologous genes should reflect the species tree accurately. This is quite plainly not the case in Figures 3 and 4. In short, a 'function' tree does not necessarily equate with an evolutionary tree.

### Toll proteins

This second case history reflects a slightly different aspect of the stereotypically differing approaches of bioinformaticians and phylogeneticists. Sequences should not be processed in a phylogenetic analysis together simply on the basis of a BLAST hit between two regions of the gene or protein. Although attractive from an automation perspective, such action leads one to blindly make the implicit assumption that the whole gene is homologous rather than just the region if identified similarity. This can ultimately lead to false phylogenies resulting from domain sharing.

*Toll* is a *Drosophila* gene that is responsible for dorsal–ventral polarity during development (Anderson *et al*., 1985) but also, intriguingly, is associated with the innate immune response of *Drosophila* (Ooi *et al*., 2002; Tauszig *et al*., 2000, 2002; Williams *et al*., 1997). Consequently from the outset this gene appears to have been involved in some quite obtuse evolutionary tinkering. The gene codes for a protein with a single transmembrane domain, an extracellular domain involved in ligand reception, and an intracellular domain involved in signal transduction. The mammalian homologue of the *Drosophila* Toll proteins, Toll-like receptors (TLR), appear to be involved specifically with innate immunity and the triggering of adaptive immunity (Medzhitov *et al*., 1997), reviewed in (Hoffman *et al*., 1999; Muzio *et al*., 2000). In vertebrates, the Toll-like receptors are directly involved with detecting ligands associated with pathogens, as outlined in Table 1. The *Drosophila* Toll receptors, on the other hand, appear to receive ligands that represent indirect signals of pathogenic presence. The Toll and Toll-like receptors also share sequence similarity with the interleukin-1 (IL-1) receptors (Gay and Keith, 1991), and their accessory proteins (Greenfeder *et al*., 1995), which constitutes part of the adaptive immune system. Consequently, there is another intriguing link, this time between innate and adaptive immune responses of vertebrates. There is also a link between the 'immune system' of plants and animals through the sequence similarity of the Toll group of genes to the disease resistance genes of plants (Whitham *et al*., 1994). Finally, a fourth set of proteins show sequence similarity to Toll, TLR and IL-R. This final group are associated with aspects of the signal transduction from the receptors and include the MyD88 protein (Horng *et al*., 2001; Bonnert *et al*., 1997) and the TIRAP protein (Horng *et al*., 2002; Yamamoto *et al*., 2002).

**Table 1.** Ligands and associated pathogens recognized by the vertebrate Toll-like receptors

| Toll-like receptor | Ligand | Pathogen |
|---|---|---|
| TLR-1 | Unknown | Unknown |
| TLR-2 | PGN | Gram-positive bacteria |
| TLR-3 | ds RNA | Viruses |
| TLR-4 | LPS | Gram-negative bacteria |
| TLR-5 | Flagellin | Flagellate bacteria |
| TLR-6 | LPS | Gram-negative bacteria |
| TLR-7 | R-837, R-848 | Viruses |
| TLR-8 | R-848 | Viruses |
| TLR-9 | CpG dinucleotiodes | Prokaryote DNA |
| TLR-10 | Unknown | Unknown |

Abbreviations: ds, double-stranded; LPS, lipopolysaccharide; PGN, peptidoglycan; TLR, toll-like receptor; R-837, imiquimod; R-848, resiquimod.
Compiled from Alexoupoulou *et al.* (2001); Hayashi *et al.* (2001); Hemmi *et al.* (2000); Takeuchi *et al.* (1999, 2001).

### The TIR domain and the extrapolated inference of homology

The proteins of Toll, TLR, IL-1R, MyD88, TIRAP, IL-1RAP and various plant resistance genes all share sequence similarity in one domain, the so-called Toll/interleukin-1R (TIR) domain. This domain is actually a module that promotes homotypic interaction between the receptors and cytoplasmic proteins of the signal cascade (Xu *et al*., 2000). Consequently, a BLAST analysis would rapidly lead to an assemblage of sequences that included members from all these groups. In order to elucidate the relationships between the various TIR containing proteins, one may be tempted to pass the sequences identified in the BLAST search straight into an alignment and tree-building algorithm. The resulting tree is shown in Figure 5. This phylogeny would lead one to conclude that the insect Toll genes form a clade with the Toll-like receptors, and that the IL-1 receptors form an outgroup. This may lead one to suppose that the components of the adaptive immune system were already in existence

Copyright © 2004 John Wiley & Sons, Ltd.

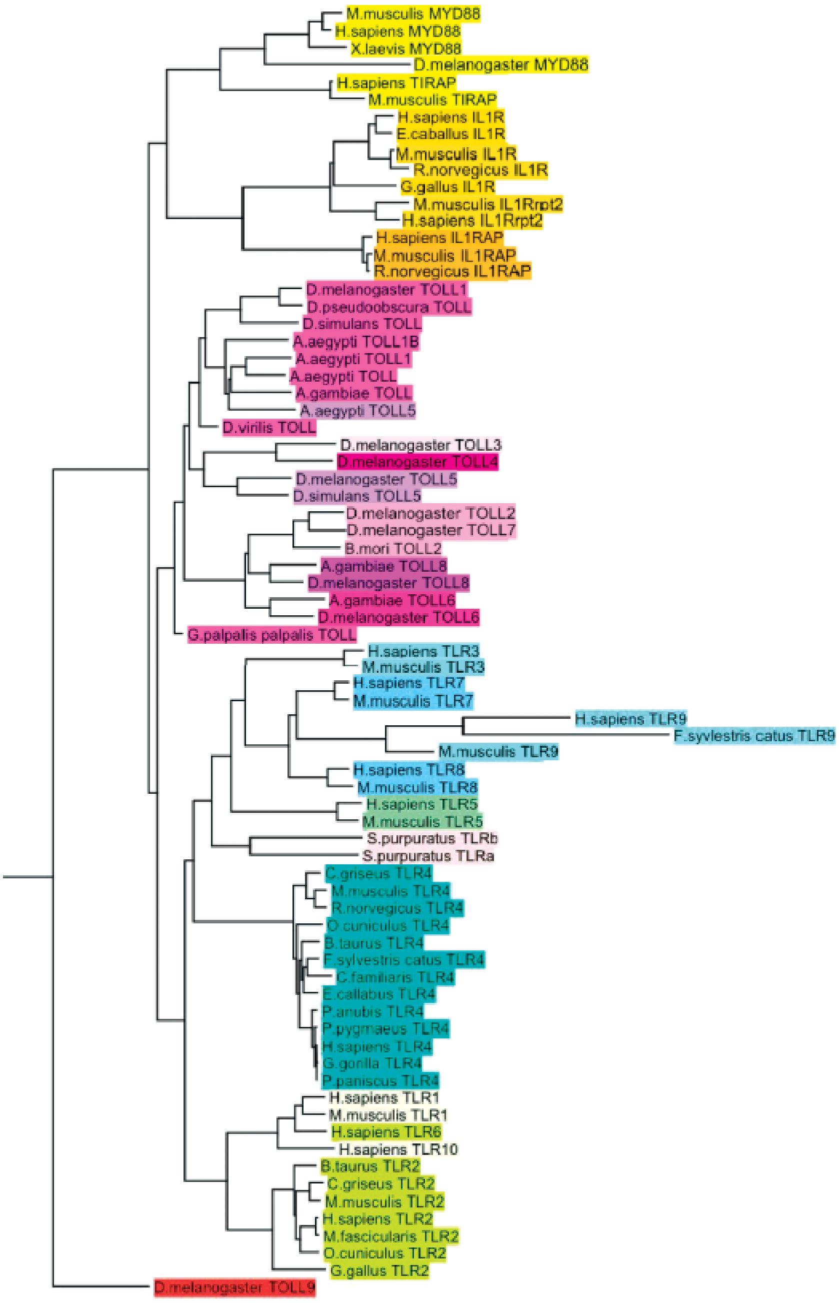

**Figure 5.** A rooted neighbour-joining tree of TIR domain containing proteins. Colour labelling of taxa is as follows: Toll proteins from dipterans (pink); vertebrate TLR (blue); MyD88 and TIRAP (yellow); IL-1R (gold); interleukin receptor accessory proteins (orange). The tree was rooted using plant resistance genes as an outgrouping clade before the divergence of vertebrate and arthropod lineages, which may lead one to ask why arthropods do not have an adaptive immune system. The adaptive immune system is thought to be about 450 million years old (Agarwal *et al.*, 1998), whereas the deuterostomian and ecdysozoan lineages, in which vertebrates and arthropods are respectively placed, diverged from each other in the Precambrian period, over 543 million years ago (Balavoine and Adoutte, 1998). Consequently, it

Copyright © 2004 John Wiley & Sons, Ltd. 

would appear that we may already be confused in our analysis.

### Homology and the TIR domain containing proteins

The use of the available *a priori* knowledge allows one to summarize the domains present within the TIR domain containing proteins, shown in Figure 6. Immediately it can be seen that the entire length of the various proteins are not homologous, or at least, their homologous nature is highly questionable. The Toll and Toll-like receptors share a similar domain structure including an extracellular leucine-rich repeat region that is involved in ligand recognition. The extracellular region of IL-1 receptors, however, is an immunoglobin module, which is more reminiscent of the adaptive immune system. The immunoglobin module is not directly comparable to the leucine-rich repeat module. In the case of the MyD88 protein, there is a death domain module present, so-called because its first identification in apoptotic proteins. Similar to the TIR module, the death domain module is a homophyllic domain facilitating protein–protein interaction. In the case of the MyD88 protein, the death domain module interacts with death domain containing proteins from the next step of the signal cascade, Pelle in the case of *Drosophila*, IRAK (IL receptor-associated kinase) and IRAK-2 in the case of vertebrates (Muzio *et al*., 1997). Consequently, it is apparent that only the TIR module is homologous between all these proteins, despite their inclusion on the basis of BLAST results.

### The TIR phylogeny

An alignment based on just the TIR domain ensures a homologous comparison of the TIR containing proteins and gives rise to the tree shown in Figure 7. Immediately, we can see that the insect Toll proteins are not placed with the vertebrate Toll-like receptors to the exclusion of the IL-1 receptors. It would appear that the formation of a single clade between the Toll and Toll-like receptors in Figure 5 is probably due to the distorting effects of domain sharing between Toll and TLR. Instead, the tree in Figure 7 is compatible with the notion that part of the IL-1 receptor was tinkered from the Toll-like receptor TIR domain. We can also see that the TIR domains of IL-1 receptor accessory proteins were probably made from a common ancestor with the IL-1 receptor TIR domains. The MyD88 and TIRAP proteins form a clade that joins the Toll-like receptor clades at a basal position. Interestingly, the TIR domains of MYD88 proteins and TIRAP proteins appear to have a common ancestor prior to the split of the arthropod and vertebrate lineages. TIRAP, however, which offers an alternative biochemical path for signal transduction from the TLR4 receptor to MyD88, has yet to be identified outside the vertebrates. It may be the case that this pathway has been lost in *Drosophila* due to the degeneracy caused by overlapping function between MyD88 and TIRAP. Naturally, this does lead one to ask why the pathway has then been maintained in vertebrates. More recently, a second adaptor molecule, TICAM has been identified which moderates the signal transduction from TLR3 (Oshiumi *et al*., 2003). Although preliminary phylogenetic analysis indicates this sequence type is probably most closely related to the TIRAP proteins, it has been excluded from this review due to low overall sequence similarity to Toll and Toll-like receptor sequences.

Perhaps the most striking feature about the tree in Figure 7 is the congruence between the TIR phylogeny and the function of the TIR containing protein. In particular, some light appears to be thrown on the evolution of the Toll-like receptors. The Toll-like receptor TIRs appear to fall into two clades, one that includes receptors that detect large molecules (TLR2, TLR4, TLR5, TLR6), and another group that detects small molecules (TLR3, TLR7, TLR8 and TLR9). The antiquity of this split is evident in the positioning of the basal node to these two clades relative to the MyD88 clade. Interestingly, this tree supports the notion that the IL-1 receptor clade TIR domains were more likely to have been tinkered from the lineage of Toll-like receptors that are involved in the small molecule

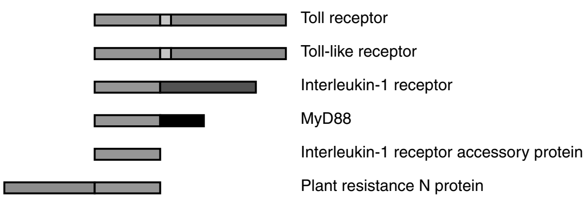

**Figure 6.** The modular structure of TIR domain containing proteins. Modules coloured as follows: white, TIR domain; grey, cysteine-rich domain; horizontal lines, immunoglobin domain; black, death domain; diagonal lines, leucine-rich repeats

Copyright © 2004 John Wiley & Sons, Ltd.

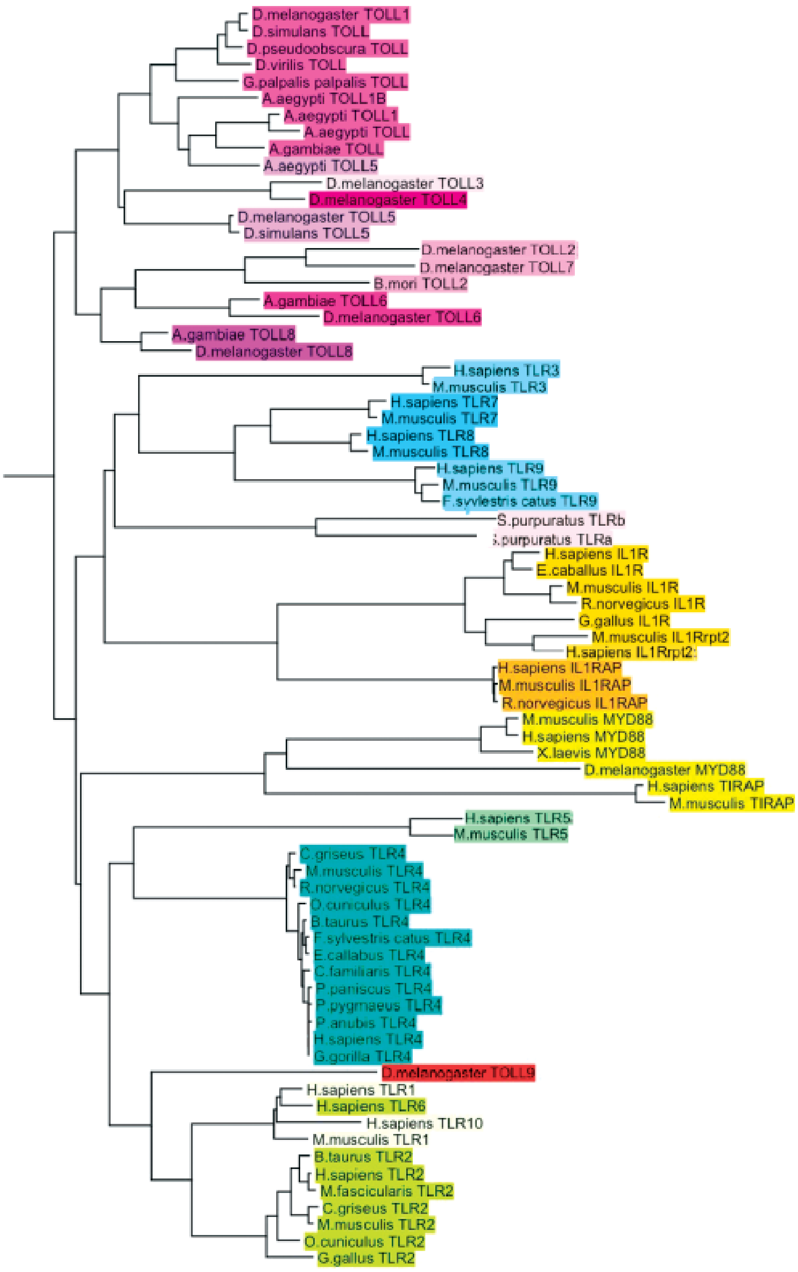

**Figure 7.** A rooted neighbour-joining tree of the TIR domains of TIR domain containing proteins. Taxa are colour-labelled as follows: various shades of red and pink, invertebrate Toll proteins; turquoise, Toll-like LPS sensitive receptors (TLR4); green, Toll-like PGN-sensitive receptors (TLR2, TLR6); birch green, Toll-like flagellin-sensitive receptors (TLR5); blue, Toll-like small molecule sensitive receptors; yellow, MyD88 and TIRAP proteins; gold, IL–1 receptors; orange, IL-1 receptor accessory proteins. The tree was rooted using the TIR domains of plant resistance genes detection. In respect to this aspect, the *sensu bifo* and the *sensu stricto* result in the same groupings, which can only serve to confuse matters. In this case the orthologous taxa do tend to have the same function also. However, similar function does not define orthology *sensu stricto*.

A second striking aspect of this tree regards the apparent antiquity of the Toll and Toll-like

Copyright © 2004 John Wiley & Sons, Ltd. 

clades. It has been suggested that the evolution of the innate immune system occurred independently in arthropods and vertebrates because Toll proteins and Toll-like proteins form separate clades (Hughes, 1998; Luo and Zheng, 1999). However, with the addition of new sequence information, it can clearly be seen that the picture is not so simple. The *Drosophila* Toll-9 receptor (Ooi *et al*., 2002) clearly upsets the phylogeny by its TIR domain forming a clade with the vertebrate Toll-like receptors TIR domains associated with detecting with detecting lipopolysaccharide (LPS) molecules. Interestingly, Toll-9 appears to be involved in antimicrobial expression in response to LPS (Ooi *et al*., 2002). The Toll-like receptor TLR-2, with which the Toll-9 forms a clade, appears to be primarily involved with detection of the peptidoglycan (PGN) ligand, but has been implicated in LPS detection also (Yang *et al*., 1998). This inevitably leads one to the question of whether the Toll-9 receptor may, atypically for the *Drosophila* Toll receptors, be directly involved with ligand detection like the Toll-like receptors. Consequently, the common ancestor of the TLR2 and TLR4 clades appears to predate the arthropod–vertebrate split.

The Toll-like receptors appear to be very much older than might have been anticipated previously. This fact is echoed in the placement of the sea urchin, *Strongylocentrotus purpuratus*, Toll-like sequences within the vertebrate Toll-like receptor clade, rather than being placed as an outgroup to it. Additionally, the recent completion of the *Ciona* genome identifies three Toll-like receptors as well as an IL-1 receptor (Dehal *et al*., 2002). The antiquity of the Toll-like receptor clades is also echoed by the MyD88 clade, which also includes a *Drosophila* sequence. The evidence appears to be mounting in favour of an ancient origin of the innate immune system prior to the protostome–deuterostome split, as opposed to an independent evolution of the system in the two lineages. In the case of the Toll-like receptors, the evidence supports an existence of three or four receptor types, two or three involved with large ligand reception and the other with small ligand reception. An ancient origin of Toll-like receptors leads one to question the absence of *Drosophila* sequences in the small ligand receptor lineages and the receptor lineages that detect large ligands other than LPS. While it appears that Toll itself only recognizes the Spaetzle ligand, many of the other Toll proteins, including Toll-9, have not had their true ligands characterized. The phylogenetic placement of Toll-9 TIR domain suggests the possibility that maybe this Toll receptor is involved with direct pathogenic ligand recognition, rather than binding the to Spaetzle ligand.

### Domain homology is not protein homology

The TIR domain shows just one history of the Toll and Toll-like proteins, and the associated proteins that also include a TIR domain. A separate analysis of the extracellular domain of the Toll and Toll-like proteins may help to shed a little more light on their history. However, a rich picture can be built up from the evidence of the TIR domain in conjunction with as much *a priori* information as possible. The conclusions reached in this analysis simply could not have been made by blindly analysing TIR domain-containing proteins on the basis of BLAST hits.

### The Bioinformatician vs. the Phylogeneticist

In order for good phylogenetic practice to be employed by bioinformaticians, it is worth highlighting the differences in practice between normal bioinformatics and phylogenetics. For the purposes of this article, the bioinformatician and the phylogeneticist have been stereotyped to illustrate the differences in their respective approaches.

Bioinformatics has developed its own culture of understanding, termed the *sensu bifo* in this article, which would not necessarily be recognized by evolutionary biologists. In particular, this article has highlighted the frequent modified use of the term ‘orthologue’ in the bioinformatics discipline. As highlighted with the *sensu bifo* use of ‘orthologue’ in the amidohydrolase case history, the modified use of such terminology can lead to the formulation of implicitly incorrect evolutionary paradigms.

The discipline of bioinformatics has evolved around its methods of data retrieval. As a consequence, bioinformaticians tend to start with huge datasets and whittle down to a small amount of data of interest. Conversely, the phylogeneticist builds up from small datasets, proceeding only with established homology, expanding to larger datasets. This aspect is exemplified in this article by the vetting

Copyright © 2004 John Wiley & Sons, Ltd.

of alignment scores in the early stages of a phylogenetic analysis, which assesses the amount of 'guesswork' genetic distance algorithms have to do in order to bridge long distances between taxa. Adjacent to this aspect is that the bioinformatician tends to expect to be able to draw inferences across huge genetic distances.

The phylogeneticist will analyse in independent units of domains, introns and exons, where as the bioinformatician will tend to analyse one level of organization higher, at the gene level. This aspect is exemplified in this article in the Toll case history in which one can produce an erroneous phylogeny from the whole gene, or one can first establish the domains that are homologous.

In contrast to the bioinformatician, the phylogeneticist will try to incorporate as much *a priori* information into the formulation of the analysis as possible. This probably represents the most significant difference between the approaches of the bioinformatician and the phylogeneticist. The difference of approach is illustrated in this article by the inclusion of a summary of domain shuffling aspects of evolutionary biology, and the need to split the data up to represent those possibilities on the basis of *a priori* information. It is undoubtedly this area that presents the greatest challenge to bioinformatics. Bioinformaticians will be familiar with the problem from completely disparate aspects of their own discipline, namely data integration.

In summary, bioinformatics deals with high-throughput systems, where there is virtually no *a priori* information other than the sequence itself, and consequently all biological input to the interpretation and contextualization of the data is *a posteriori*. To put it another way, effects, biological realities, are interpreted by causes, bioinformatic processes. As a high-throughput system, until data integration is effectively achieved, the biological contextualization of the data will tend to be of an *a posteriori* nature. The process of phylogenetics is *a priori* in its biological contextualization, e.g. one must know which characters are homologous before one starts an analysis. Obviously, there is some *a posteriori* reasoning of effects, evolutionary history, to causes, tree-building processes, which are firmly based on *a priori* assumptions. In the long term it would be desirable to bring phylogenetics to a high-throughput environment, to which end the development and useful implementation of data integration needs to be realized. This, in effect, would increase the *a priori* input into the analysis, but the bioinformatician would be able to approach the analysis in an *a posteriori* way. However, until that time, and before that time can be reached, *a priori* data should be utilized in phylogenetic analysis and incorporated into the bioinformatician's culture of understanding.

### Concluding remarks

A culture of understanding leading to a culture of practice in bioinformatics has been tempered by a need to trawl through vast datasets. The extent of genomic data generation has now reached the point at which questions of a phylogenetic nature are being asked, e.g. regarding the apparent absence of orthologues, which cannot be simply assigned to missing data but seem more likely to have an explanation rooted in evolutionary biology. Consequently, in order for bioinformatics to address these types of questions, it needs to examine its own approaches and starting assumptions.

### Acknowledgements

Thanks to Paul Dodson and David Tilley for useful discussions. Also thanks to the two anonymous referees for useful amendments to the manuscript.

### References

Agarwal A, Eastman QM, Schatz DG. 1998. Transposition mediated by RAG1 and RAG2 and its implications for the evolution of the immune system. *Nature* **394**: 744.

Alexopoulou L, Holt AC, Medzhitov R, Flavell RA. 2001. Recognition of double-stranded RNA and activation of NF-$\kappa$B by Toll-like receptor 3. *Nature* **413**: 732–738.

Allaby RG, Brown TA. 2001. Network analysis provides insights into evolution of 5S rDNA arrays in *Triticum* and *Aegilops*. *Genetics* **157**: 1331–1341.

Anderson KV, Jurgens G, Nusslein-Volhard C. 1985. Establishment of the dorsal-ventral 1 polarity in the *Drosophila* embryo: genetic studies on the role of the Toll gene product. *Cell* **42**: 779–789.

Balavoine G, Adoutte A. 1998. One or three Cambrian radiations? *Science* **280**: 397.

Bockaert J, Pin JP. 1999. Molecular tinkering of G protein-coupled receptors: an evolutionary success. *EMBO J* **18**: 1723–1729.

Bonnert TP, Garka KE, Parnet P, *et al.* 1997. The cloning and characterisation of human MyD88: a member of an IL-1 receptor related family. *FEBS Lett* **402**: 81–84.

Boundy-Mills KL, de Souza ML, Mandelbaum RT, Wackett LP, Sadowsky MJ. 1997. The *atz*B gene of *Pseudomonas* sp. Strain

Copyright © 2004 John Wiley & Sons, Ltd. 

ADP encodes the second enzyme of a novel atrazine degradation pathway. *Appl Environ Microb* **63**: 916–923.

Copley RR, Bork P. 2000. Homology among $(\beta\alpha)_8$ barrels: implications for the evolution of metabolic pathways. *J Mol Biol* **303**: 627–640.

Dehal P, Satou Y, Campbell RK, *et al*. 2002. The draft genome of *Cona intestinalis*: insights into chordate and vertebrate origins. *Science* **298**: 2157–2167.

de Souza M, Sadowsky MJ, Wackett LP. 1996. Atrazine chlorohydrolase from *Pseudomonas* sp. ADP: gene sequence, enzyme purification, and protein characterisation. *J Bacteriol* **178**: 4894–4900.

Eaton RW, Karns JS. 1991. Cloning and analysis of *s*-triazine catabolic genes from *Pseudomonas* sp. strain NRRLB-12227. *J Bacteriol* **173**: 1215–1222.

Felsenstein J. 1989. PHYLIP — Phylogeny Inference Package (version 3.2). *Cladistics* **5**: 164–166.

Gay NJ, Keith FJ. 1991. *Drosophila* Toll and IL-1 receptor. *Nature* **351**: 355–356.

Goodrich JA, Lykins BW, Clark RG. 1991. Drinking water from agriculturally contaminated ground water. *J Environ Qual* **20**: 707–717.

Greenfeder SA, Nunes P, Kwee L, *et al*. 1995. Molecular cloning and characterisation of a second subunit of the interleukin receptor complex. *J Biol Chem* **270**: 13 757–13 765.

Hayashi F, Smith KD, Ozinsky A, *et al*. 2001. The innate immune response to bacterial flagellin is mediated by Toll-like receptor 5. *Nature* **410**: 1099–1103.

Hemmi H, Kaisho T, Takeuchi O, *et al*. 2002. Small antiviral compounds activate immune cells *via* the TLR7 MyD88-dependent signalling pathway. *Nature Immunol* **3**: 196–200.

Hemmi H, Takeuchi O, Kawai T, *et al*. 2000. A Toll-like receptor recognizes bacterial DNA. *Nature* **408**: 740–745.

Hoffmann JA, Kafatos FC, Janeway CA Jr, Ezekowitz RAB. 1999. Phylogenetic perspectives in innate immunity. *Science* **284**: 1313–1318.

Holm L, Sander C. 1997. An evolutionary treasure: unification of a broad set of amidohydrolases related to urease. *Proteins Struct Funct Genet* **28**: 72–82.

Horng T, Barton GM, Flavell RA, Medzhitov R. 2002. The adaptor molecule TIRAP provides signalling specificity for Toll-like receptors. *Nature* **420**: 329–333.

Horng T, Medzhitov R. 2001. *Drosophila* MyD88 is an adaptor in the Toll signalling pathway. *Proc Natl Acad Sci USA* **98**: 12 654–12 658.

Howard AD, McAllister G, Feighner SD, *et al*. 2001. Orphan G-protein-coupled receptors and natural ligand discovery. *Trends Pharm Sci* **22**: 132–140.

Hughes AL. 1998. Protein phylogenies provide evidence of a radical discontinuity between arthropod and vertebrate immune systems. *Immunogenetics* **47**: 283–296.

Hughes AL, Yeager M. 1999. Coevolution of the mammalian chemokines and their receptors. *Immunogenetics* **49**: 115–124.

Huson DH. 1998. SplitsTree: analyzing and visualizing evolutionary data. *Bioinformatics* **14**: 68–73.

Jacob F. 1977. Evolution and tinkering. *Science* **196**: 1161–1166.

Jukes TH, Cantor CR. 1969. Evolution of protein molecules. In *Mammalian Protein Metabolism*, Munro HN (ed.). Academic Press: New York; 21–132.

Kim G-J, Kim H-S. 1998. Identification of the structural similarity in the functionally related amidohydrolases acting on the cyclic amide ring. *Biochem J* **330**: 295–302.

Li W-H, Gu Z, Wang H, Nekrutenko A. 2001. Evolutionary analyses of the human genome. *Nature* **409**: 847–849.

Luo C, Zheng L. 1999. Independent evolution of *Toll* and related genes in insects and mammals. *Immunogenetics* **51**: 92–98.

Maddison DR, Maddison WP. 2002a. *MacClade 4.05 Analysis of Phylogeny and Character Evolution*. Sinauer Associates: Sunderland, MA.

Maddison WP, Maddison DR. 2002b. Mesquite: a modular system for evolutionary analysis. Version 0.992. **http://mesquiteproject.org**.

Marcotte EM, Pellegrini M, Ng H-L, *et al*. 1999. Detecting protein function and protein–protein interactions from genome sequences. *Science* **285**: 751–753.

McGuire G, Wright F. 1998. TOPAL: recombination detection in DNA and protein sequences. *Bioinformatics* **14**: 219–220.

Medzhitov R, Preston-Hurlburt P, Janeway CA. 1997. A human homologue of the *Drosophila* Toll protein signals activation of adaptive immunity. *Nature* **388**: 394–397.

Mulbry WW. 1994. Purification and characterisation of an inducible *s*-triazine hydrolase from *Rhodococcus corallinus* NRRL B15444R. *Appl Environ Microb* **60**: 613–618.

Murphy PM, Baggiolini M, Charo IF, *et al*. 2000. International Union of Pharmacology. XXII. Nomenclature for chemokine receptors. *Pharmacol Rev* **52**: 145–176.

Muzio M, Ni J, Feng P, Dixit VM. 1997. IRAK (Pelle) family member IRAK-2 and myD88 as proximal mediators of IL-1 signalling. *Science* **278**: 1612–1615.

Muzio M, Polentarutti N, Bosiso D, Prahladan MKP, Mantovani A. 2000. Toll-like receptors: a growing family of immune receptors that are differentially expressed and regulated by different leukocytes. *J Leuk Biol* **67**: 450–456.

Nagy I, Compernolle F, Ghys K, Vanderleyden J, de Mot R. 1995. A single P-450 system is involved in degradation of the herbicides EPTC (*S*-ethyl dipropylthiocarbamate) and atrazine by *Rhodococcus* sp. strain NI86/21. *Appl. Environ Microb* **61**: 2056–2060.

Nurminsky DI, Nurminskaya MV, de Aguiar D, Hartl D. 1998. Selective sweep of a newly evolved sperm-specific gene in *Drosophila*. *Nature* **396**: 572–575.

Ooi JY, Yagi Y, Hu X, Ip YT. 2002. The *Drosophila* Toll-9 activates a constitutive antimicrobial defense. *EMBO Rep* **3**: 82–87.

Oshiumi H, Matsumoto M, Funami K, *et al*. 2003. TICAM-1, an adaptor molecule that participates in Toll-like receptor 3-mediated interferon-$\beta$ induction. *Nature Immunol* **4**: 161–167.

Page RDM. 1995. Parallel phylogenies: reconstructing the history of host–parasite assemblages. *Cladistics* **10**: 155–173.

Page RDM. 1998. GeneTree: comparing gene and species phylogenies using reconciled trees. *Bioinformatics* **14**: 819–820.

Patthy L. 1999. Genome evolution and the evolution of exon-shuffling — a review. *Gene* **238**: 103–114.

Posada D, Crandall KA. 2001. Evaluation of methods for detecting recombination from DNA sequences: computer simulations. *Proc Natl Acad Sci USA* **98**: 13 757–13 762.

Piutti S, Semon E, Landry D, *et al*. 2003. Isolation and characterization of *Nocardioides* sp. SP12, an atrazine-degrading bacterial strain possessing the gene trzN from bulk and maize rhizosphere soil. *FEMS Microb Lett* **221**: 111–117.

Copyright © 2004 John Wiley & Sons, Ltd. 

Robertson DL, Sharp PM, McCutchen FE, Hahn BH. 1995. Recombination in HIV-1. *Nature* **374**: 124–126.

Sadowsky MJ, Tong Z, de Souza M, Wackett LP. 1998. AtzC is a new member of the amidohydrolase protein superfamily and is homologous to other atrazine-metabolizing enzymes. *J Bacteriol* **180**: 152–158.

Seffernick JL, Chapir N, Schoeb M, *et al*. 2002. Enzymatic degradation of chlorodiamono-*S*-triazine. *Appl Environ Microb* **68**: 4672–4675.

Seffernick JL, de Souza ML, Sadowsky MJ, Wackett LM. 2001. Melamine deaminase and atrazine chlorohydrolase: 98% identical but functionally different. *J Bacteriol* **183**: 2405–2410.

Shields DC. 2000. Gene conversion among chemokine receptors. *Gene* **246**: 239–245.

Shao ZQ, Behki R. 1995. Cloning of the genes for degradation of the herbicides EPTC (*S*-ethyl dipropylthiocarbamate) and atrazine from *Rhodococcus* sp. strain TE1. *Appl Environ Microb* **61**: 2061–2065.

Shao ZQ, Seffens W, Mulbry W, Behki RM. 1995. Cloning and expression of the *S*-triazine hydrolase gene (*trzA*) from *Rhodococcus corallinus* and development of the *Rhodococcus* recombinant strains capable of dealkylating and dechlorinating the herbicide atrazine. *J Bacteriol* **177**: 5748–5755.

Shimizu N, Gojobori T. 2000. How can human and simian immunodeficiency viruses utilise chemokine receptors as their coreceptors? *Gene* **259**: 199–205.

Storm CEV, Sonnhammer ELL. 2001. NIFAS: visual analysis of domain evolution in proteins. *Bioinformatics* **17**: 343–348.

Takeuchi O, Hoshino K, Kawai T, *et al*. 1999. Different roles of TLR2 and TLR4 in recognition of Gram-negative and Gram-positive bacterial cell wall components. *Immunity* **11**: 443–451.

Takeuchi O, Kawai T, Mühlradt PF, *et al*. 2001. Discrimination of bacterial lipoproteins by Toll-like receptor 6. *Int Immunol* **13**: 933–940.

Tauszig-Delamasure S, Bilak H, Capovilla M, Hoffmann JA, Imler JL. 2002. *Drosophila* MyD88 is required for the response to fungal and Gram-positive bacterial infections. *Nature Immunol* **3**: 91–97.

Tauszig S, Jouanguy E, Hoffmann JA, Imler JL. 2000. Toll-related receptors and the control of antimicrobial peptide expression in *Drosophila*. *Proc Natl Acad Sci USA* **97**: 10 520–10 525.

Topp E, Mulbry WM, Zhu H, Nour SM, Cuppels D. 2000. Characterisation of *S*-triazine herbicide metabolism by a *Nocardioides* sp. isolated from agricultural soils. *Appl Environ Microb* **66**: 3134–3141.

Whitham S, Dinesh-Kumar SP, Choi D, *et al*. 1994. The product of the tobacco mosaic virus resistance gene N: similarity to toll and the interleukin-1 receptor. *Cell* **78**: 1101–1115.

Williams MJ, Rodriguez A, Kimbrell DA, Eldon ED. 1997. The 18-wheeler mutation reveals complex antibacterial gene regulation in *Drosophila* host defense. *EMBO J* **16**: 6120–6130.

Xu Y, Tao X, Shen B, *et al*. 2000. Structural basis for signal transduction by the Toll/interleukin-1 receptor domains. *Nature* **408**: 111–115.

Yamamoto M, Sato S, Hemmi H, *et al*. 2002. Essential role for TIRAP in activation of the signalling cascade shared by TLR2 and TLR4. *Nature* **420**: 324–329.

Yang R-B, Mark M, Gray A, *et al*. 1998. Toll-like receptor-2 mediates lipopolysaccharide-induced cellular signalling. *Nature* **395**: 284–288.

Yuan G, Bin JC, McKay DJ, Snyder FF. 1999. Cloning and characterisation of human guanine deaminase. *J Biol Chem* **274**: 8157–8180.

Zischler H, Geisert H, von Haesseler A, Pääbo S. 1995. A nuclear ‘fossil’ of the mitochondrial D-loop and the origin of modern humans. *Nature* **378**: 489–492.

Zuany-Amorim C, Hastewell J, Walker C. 2002. Toll-like receptors as potential therapeutic targets for multiple diseases. *Nature Rev* **1**: 797–807.

Zullo S, Sieu LL, Slightom JL, Hadler HI, Eisenstadt JM. 1991. Mitochondrial D-loop sequences are integrated in the rat nuclear genome. *J Mol Biol* **221**: 1223–1235.

Copyright © 2004 John Wiley & Sons, Ltd. 
